# Dose calculation accuracy of lung planning with a commercial IMRT treatment planning system

**DOI:** 10.1120/jacmp.v4i4.2505

**Published:** 2003-09-01

**Authors:** Patrick N. McDermott, Tongming He, A. DeYoung

**Affiliations:** ^1^ Department of Radiation Oncology, Karmanos Cancer Institute Wayne State University Detroit Michigan 48201

**Keywords:** IMRT, lung cancer, Monte Carlo, dosimetry

## Abstract

The dose calculation accuracy of a commercial pencil beam IMRT planning system is evaluated by comparison with Monte Carlo calculations and measurements in an anthropomorphic phantom. The target volume is in the right lung and mediastinum and thus significant tissue inhomogeneities are present. The Monte Carlo code is an adaptation of the msnp code and the measurements were made with TLD and film. Both the Monte Carlo code and the measurements show very good agreement with the treatment planning system except in regions where the dose is high and the electron density is low. In these regions the commercial system shows doses up to 10% higher than Monte Carlo and film. The average calculated dose for the CTV is 5% higher with the commercial system as compared to Monte Carlo.

PACS number(s): 87.53.‐j, 87.66.‐a

## I. INTRODUCTION

The purpose of this study is to evaluate the accuracy of doses calculated by an IMRT planning system in the presence of inhomogeneities and under clinically relevant conditions. We have made comparisons in the thorax of an anthropomorphic phantom between the dose distribution computed by an IMRT treatment planning system (Corvus v4.6, NOMOS Corp., Cranberry Township, PA), measured doses and Monte Carlo (an adaptation of msnp) calculated doses. The measurements were made with film and thermoluminescent dosimeters (TLD). A comparison of this study with previously published work of a similar nature is discussed in Sec. VII.

Most clinical physicists carry out patient specific IMRT validation measurements in slab phantoms which are homogeneous. It is known that accurate dose computation in the presence of large tissue inhomogeneities is challenging and that many algorithms exhibit shortcomings in this domain.^1^ Furthermore, accurate dose calculation may be more crucial for inversely planned IMRT than for conventional treatment planning because plan optimization is based on the calculated dose matrix.^2^ For these reasons it is important to assess the dose calculation accuracy of IMRT treatment planning systems under conditions of realistic geometry and composition.

## II. METHODS AND MATERIALS

**Figure 1:**
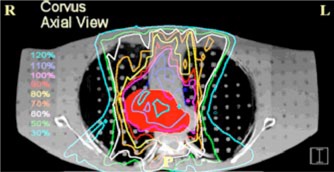
(Color) Shows an axial cross section CT image of the anthropomorphic phantom used in this study. The grid of plugs that can be removed and replaced with TLDs is evident. The plugs are approximately 1.5 cm apart. The region colored red is the CTV. The dose distribution for the Corvus plan is shown with full correction for inhomogeneities.

**Figure 2:**
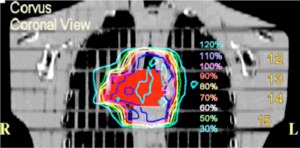
(Color) A coronal view of the same treatment plan shown in The red structure is the CTV. The phantom slab numbers are displayed on the right‐hand side of the figure.

We have used the anthropomorphic phantom with the trade name RANDO (The Phantom Laboratory, Salem, NY). This phantom was fabricated using a tissue equivalent resin (ρ=0.985 g/cm^3^) molded around a natural skeleton. The phantom has air cavities (pharynges, larynx, trachea, stem bronchi, etc.) which were made from impressions taken from a cadaver. RANDO has realistic lungs with a density of 0.32 g/cm^3^. RANDO is sectioned axially into 2.5 cm thick slabs (see Figs. 1 and 2). Film can be placed between slabs for dosimetry studies. Within each slab there are plugs arranged in a grid with a spacing of 1.5 cm between adjacent plugs (see Fig. 1). These plugs may be removed and replaced by TLDs.

Three fiducial markers (2 mm diameter lead BBs) were placed on the phantom prior to a CT scan, one each on the right lateral, left lateral, and anterior surfaces. Paper spacers were placed between the slabs, where it was anticipated that film would be positioned, in order to preserve geometric spacing between slabs. The relationship between CT number and relative electron density was calibrated with a Gammex rmi 467 CT electron density phantom (Gammex rmi, Middleton, WI). The slice thickness and table feed were 4 mm. The scan extended from a point superior to the apex of the lungs to inferior of the diaphragm. Each CT slice contains 512 by 512 pixels. Each pixel is about 1 mm in size.

The IMRT treatment planning system is Corvus (v4.6) produced by the NOMOS corporation. The dose calculation is based on a finite size pencil beam algorithm with a beamlet size of 1 cm by 1 cm. The tissue inhomogeneity correction is based on a path length correction for each pencil beam. The linac used for delivery is a Varian Clinac 2300 C/D with 52 leaves (26 pairs) (Varian Medical Systems, Inc., Palo Alto, CA). Leaf width is 1.0 cm projected to isocenter The beam energy is 6 MV

The target was contoured by a physician in Corvus. It is intended to be representative of a typical lung tumor target volume (see Figs. 1 and 2). The target occupies a portion of the mediastinum and right lung. The volume of the clinical target volume (CTV) is 148 cm^3^. The portion of the target in the lung has electron density values characteristic of lung tissue. A real tumor may have higher electron density values. This is a caveat that must be considered when interpreting our results. The organs at risk that were contoured are the left lung (2300 cm^3^), right lung (2500 cm^3^), and the spinal cord. Overlapping structures are not permitted in Corvus and therefore the contoured right lung does not include the portion of the target that is in the right lung. Margins for the expansion of the CTV to the PTV are 11 mm in both the anterior and posterior direction, 14 mm in both the right and left direction, and 18 mm in both the superior and inferior direction.^3^

The Monte Carlo software is an adaptation of the msnp 4B code.^4^ Validation measurements for this adaptation were performed in both homogeneous and heterogeneous phantoms and are described in detail by He.^5^ Dose was calculated in 3 mm×3 mm×3 mm voxels with statistical uncertainties less than 1%.

The goal dose for the target is 180 cGy. We were restricted to axial beams because the msnp calculations are only possible for axial beams due to current software limitations. For these reasons the plan is not the best possible plan, and perhaps not even a good plan, but it is not unrealistic. Our focus here is not on the quality of the plan, but on the accuracy of the dose calculation. A total of five beams were used: an anterior beam (gantry angle 0°), two anterior oblique beams (25° and 341°), and two posterior oblique beams (127° and 225°). All gantry angles are based on the International Electrotechnical Commission (IEC) scale. The optimization was performed with inhomogeneity corrections turned on. An axial view of the dose distribution is shown in Fig. 1 and a coronal view in Fig. 2. The Corvus voxel size for dose calculation is 1 mm×1 mm×1 mm.

Ready pack $10^{\prime \prime }\times 12^{\prime \prime }$ Kodak EDR2 film (Kodak, Rochester, NY) was placed in between slabs 12 and 13, 13 and 14, and lastly 14 and 15 (see Fig. 2). Three widely spaced pinholes were made in each film at known plug positions for later image registration. The phantom was set up in the treatment room using the fiducial markers that were placed prior to the CT scan. Before delivering the irradiation, anterior and lateral orthogonal films were exposed. These films were compared to DRRs generated by Corvus to ensure proper positioning. Small adjustments in the position of the phantom were made as a result of this procedure. A film calibration curve was constructed using an MLC step wedge previously calibrated with an ion chamber. Eleven data points were used to define the H&D curve with doses between 0 and 350 cGy. All films were taken from the same batch and processed simultaneously. The films were scanned with a 16 bit CCD film scanner (Vidar VXR‐16DP, Vidar Systems Corp, Herndon, VA). The scanning parameters were 357 μm resolution and 3 by 3 median filtering. This provides resolution of 1 mm or better. The isodose lines from the film were superimposed on the Corvus treatment planning images (see Sec. IV).

van Dyk has proposed criteria for the accuracy of dose calculations for a composite anthropomorphic phantom.^6^ The criterion used in a specific region depends on whether the dose is high or low there and on whether the dose gradient is high or low. The criteria are based on one standard deviation. This means that 68% of comparisons should lie within the stated tolerance. In a high dose region where the dose gradient is low, the dose accuracy at corresponding locations should be 4%. In high dose gradient regions (>30%/cm) the distance to agreement criterion is used. The distance to agreement is the distance between a point with a specific dose value and the nearest point in the comparison dose distribution that has the same dose value. In such regions the distance to agreement should be no more than 3 mm. In low dose regions (<7% of the normalization dose) where the dose gradient is also low, the dose agreement should be within 3%.

It is difficult to make experimental dose measurements in an anthropomorphic phantom with the accuracy necessary to discern these distinctions. The error in the registration of the film to the treatment planning dose distribution would need to be <3 mm to clearly demonstrate a distance to agreement of 3 mm. This is difficult to accomplish. In high dose, low gradient regions, dose measurements would need to be accurate to considerably better than 4% to discern discrepancies on that level. This is again a difficult proposition in that the accuracy of TLD measurements is on the order of 3%.^7^ Dose distributions on Corvus can only be displayed for axial, sagittal, and coronal planes. The film plane was not precisely parallel to the CT axial plane. There was an anterior to posterior tilt of approximately 1°. Three pin pricks were made in each film on the left, right, and anterior for later registration with treatment planning images. It is estimated that the film registration accuracy is approximately 3 mm. Dosimetric accuracy of 2%–3% seems possible with EDR2 film.^8^ ^,^ ^9^ The irradiation setup uncertainty, even with a rigid stationary phantom, may be on the order of 1–2 mm despite the use of localization films. On the slices that most closely correspond to the film location, adjacent 10% Corvus decrement isodose lines sometimes touch and even cross one another. As an example, in Fig. 5(b) the Corvus 162 cGy line actually crosses the Corvus 144 cGy line in the right posterior lung where the gradient is very steep. In view of all of these considerations it is evident that the van Dyk criteria are difficult to use in practice because the tolerance level is on the same order, or perhaps even less, than the measurement uncertainties. These considerations do not apply, however, to the comparison between Corvus and msnp. For this comparison the dose distributions can be very accurately registered.

**Table I:** CTV statistics.

	msnp	Corvus	Corvus uncorrected
Volume (cm^3^)	147.5	148.4	148.4
Percent volume below goal dose	20	5	60
Minimum dose (% of goal)	69	75	61
Maximum dose (% of goal)	130	127	116
Mean dose (% of goal)	107	112	97

## III. COMPARISON OF TREATMENT PLAN STATISTICS

Three treatment plans have been generated, and these are compared in this section. Two of these are Corvus plans and one is a msnp plan. In the “corrected” Corvus plan the dose optimization and the final calculated dose are based on dose computed with corrections for inhomogeneities (see Figs. 1 and 2). The normalization for the corrected plan has been chosen so that 5% of the CTV volume is below the goal dose of 180 cGy. The fluence map for this plan was taken and used to recalculate the dose in the absence of corrections. This is the Corvus “uncorrected plan.” The MU for each port was identical for each Corvus plan. The fluence delivery is precisely the same in each case, the only difference is whether corrections are made in the final dose calculation. This will show the effect of the corrections on the dose distribution.

For the msnp plan, the intensity maps were obtained from the leaf sequencing files generated by Corvus. Corvus (v4.6) does not recompute the dose after leaf sequencing and this may lead to some small differences between the msnp computed dose and the Corvus (preleaf sequencing) computed dose. The target and the normal structure contours were transferred via RTOG export from Corvus to the msnp code.

**Figure 3:**
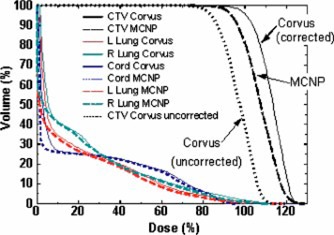
(Color) Cumulative dose volume histograms for the CTV, right and left lung, and the spinal cord. The Corvus (uncorrected) DVH represents the dose in a Corvus plan which is uncorrected for inhomogenieties. The msnp curves are based on Monte Carlo dose calculations. The corrected Corvus CTV doses are higher than the msnp doses although the minimum and maximum dose do not differ significantly. The DVHs for the organs at risk do not differ significantly.

The three plans are summarized and compared in Tables I and II and in Fig. 3. Table I lists statistics for the CTV. Values of the CTV volume are quoted to show that contour transfer from Corvus to msnp was accomplished without distortion. A comparison of the uncorrected Corvus plan with the corrected plan shows that the inhomogeneity effects are substantial. For the Corvus corrected plan the minimum dose is about 135 cGy and the maximum is about 229 cGy. In the uncorrected plan, 60% of the target volume is below the goal dose of 180 cGy, the minimum dose is about 110 cGy and the maximum dose is 209 cGy. The mean dose is approximately 15% lower for the uncorrected plan. The differences are due to the extra attenuation (in the uncorrected plan) associated with the assumption of unit relative electron density throughout the volume of the lungs. The msnp doses in the target are generally lower than computed by the corrected Corvus plan; the mean dose is approximately 5% lower, although the maximum dose is about the same. The percentage of the volume below the goal dose is 20% for msnp in comparison to only 5% for Corvus.

**Table II:** Normal structure statistics for 34 fractions. (This corresponds to a dose of 6120 cGy delivered to the CTV.)

Structure		msnp	Corvus	Corvus uncorrected
L Lung	Percent of volume above 3000 cGy	13.5	15	12.6
	Total volume (cm^3^)	2294	2296	2296
R Lung	Percent of volume above 3000 cGy	15.0	15.0	13.1
	Total volume (cm^3^)	2490	2493	2493
Cord	Percent of volume above 4500 cGy	8.5	8.8	4.7
	Maximum dose (%)	97	97	87
	Total volume (cm^3^)	79.5	82.2	82.2

Table II lists dose volume statistics for the normal structures for a 34 fraction treatment which delivers a total dose of 6120 cGy to the CTV. There is little difference between msnp and Corvus (corrected).

Figure 3 shows the cumulative dose volume histogram (DVH) for all anatomical structures for the Corvus corrected plan and for the msnp plan. Also shown in the same figure is the Corvus uncorrected CTV DVH. For the target, the msnp DVH is lower by a (possibly) significant amount. Corvus shows a somewhat higher volume of right lung at the highest doses, just as for the CTV

## IV. COMPARISON OF ISODOSE CURVES

**Figure 4:**
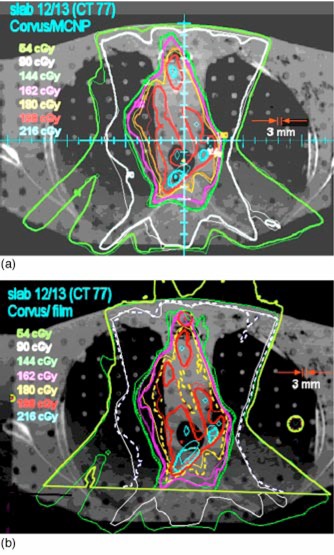
(Color) (a) Superposition of Corvus and msnp dose calculation at the location of the interface between slabs 12 and 13 of RANDO. The thick lines are msnp and the thin lines are Corvus. The agreement is excellent, so much so that in many locations it is not possible to see the superposition because isodose curves lie on top of one another. The exception is in the medial aspect of the right lung. msnp lines appear to be pushed in compared to Corvus. This indicates that Corvus doses tend to be higher in this region. (b) Isodose lines from film (thick lines, sometimes dashed) superimposed on the dose distribution calculated by Corvus for slabs 12 and 13. The 54 cGy film isodose curve extends above the anterior surface of the phantom because the film protruded above the surface. The circles are the locations of the pin pricks in the film. Registration accuracy between the film and the Corvus image is expected to be on the order of 3 mm. The agreement between the film and Corvus is good except in the posterior mediastinum and the medial aspect of the right lung. The film dose is somewhat higher in the posterior mediastinum and lower in the right lung (as is msnp).

The dose distribution on an axial slice between slabs 12 and 13 (see Fig. 2) calculated by Corvus is superimposed on the msnp dose distribution in Fig. 4(a). The agreement is excellent except in the medial aspect of the right lung, where the dose is high and the electron density is low. The msnp dose is as much as 10% lower than the Corvus dose. The dose distribution measured on film between slabs 12 and 13 is displayed superimposed on the Corvus dose distribution in Fig. 4(b). The circles in the anterior mediastinum and in the left lung are from the pin holes in the film. The measured dose distribution is therefore unreliable in the vicinity of these holes. The dose distribution on the film extends above the anterior surface of the phantom because the film projected above this surface. There are discrepancies in the same region as in Fig. 4(a). In this region the dose to the film is lower than the Corvus doses just as for the msnp doses. There are locations where the corresponding isodose lines are more than 3 mm apart.

**Figure 5:**
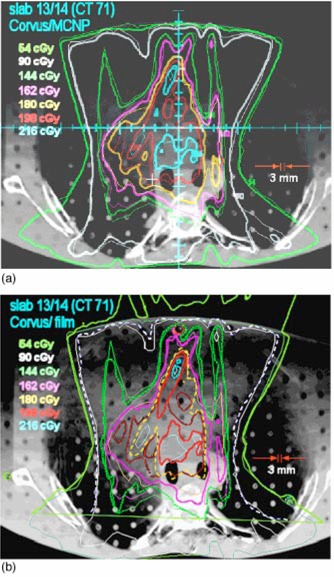
(Color) (a) Corvus isodose lines superimposed on the msnp isodose lines for slabs 13 and 14. This is a plane near the center of the dose distribution. The thin lines represent the Corvus dose distribution. This plane shows some of the largest discrepancies between Corvus and msnp which occur in the high dose low electron density region (medial aspect of the right lung). The Corvus doses are as much as 10% higher than msnp. A left/right dose profile through the cross hair is shown in (b) Corvus isodose lines superimposed on isodose lines measured with film. The thin isodose lines were computed by Corvus; the thick lines represent film measurements. On this plane we see the largest differences between Corvus and the measured doses.

**Figure 6:**
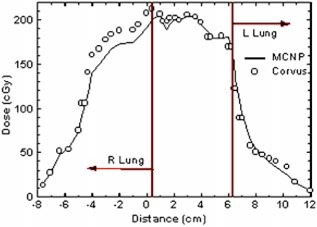
(Color) A left/right profile in the axial plane between slabs 13 and 14 [see The position of the origin is depicted as a crosshair in Corvus overestimates the dose in the right lung where the dose is high and the electron density is low.

The interface between slabs 13 and 14 (see Fig. 2) is in the middle of the high dose region. The Corvus dose distribution is superimposed on the msnp dose distribution in Fig. 5(a). The low dose lines correspond with one another fairly closely. In the right lung and mediastinum and generally throughout the region of low electron density which is occupied by the target, there are some significant discrepancies. In this region the msnp dose is lower than the Corvus dose by 10% or more. The same features are seen in Fig. 5(b), which shows the film dose measurements superimposed on the Corvus dose distribution. Figure 6 shows a profile in the left/right direction through the crosshair shown in Fig. 5(a).

**Figure 7:**
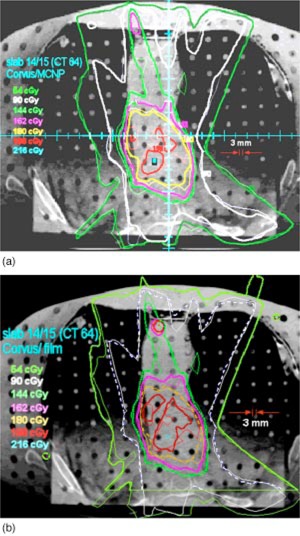
(Color) (a) The Corvus isodose distribution is superimposed on msnp on the plane between slabs 14 and 15. The thick lines are msnp. (b) The isodose curves from film measurements superimposed on the Corvus isodose curves. The thick lines are from measurement and the thin lines are from Corvus.

The interface between slabs 14 and 15 is near the inferior border of the high dose region (see Fig. 2). The msnp dose distribution is superimposed on the Corvus dose distribution in Fig. 7(a) and the film dose is superimposed on the Corvus dose in Fig. 7(b). The agreement is seen to be fairly good. The doses tend to be lower than the Corvus doses.

On the whole, the film and the msnp doses tend to be lower than the Corvus dose. This is consistent with the results presented in Sec. III which suggest that the mean Corvus dose delivered to the CTV may be 5% high. The regions in which the discrepancies are greatest are the locations where the dose is highest and the electron density is lowest. This is in the medial aspect of the right lung [see Figs. 5(a) and 5(b)].

## VI. TLD MEASUREMENTS

Twenty four TLD powder capsules (LiF TLD‐ 100, 30 mg) from the same batch were used inside RANDO slabs 13 and 14 (see Fig. 2). The powder capsules are 16 mm long and 4 mm in diameter (slightly smaller than the 5 mm diameter of the holes). The capsules were placed in the inferior portion of the cavities. Rolled paper spacers were placed in the superior portion of each cavity to hold the TLD capsules in place and to fill the air cavity. The positions of the TLDs were chosen to be in the expected high dose region and in regions of relatively low dose gradient. If the dose gradient across a capsule is large, then interpretation of the reading is problematic. Ten TLD capsules were used as standards. The standards were given a dose of $D_{0}=180$ cGy. The average reading for the ten standards was ${\bar{Q}}_{0}=15.33$ μC and the standard deviation is $\sigma_{0}=0.53$ σC (3.5%). We assume that this is characteristic of this batch of TLDs. The relatively large standard deviation limits the accuracy to which the Corvus and the Monte Carlo dose calculations can be tested. It is estimated that the uncertainty (one standard deviation) in the dose *D* measured by a TLD is:

(1) $$\sigma_{D}=\sigma_{0}D{\left(\frac{1}{n{\bar{Q}}_{0}^{2}}+\frac{1}{Q^{2}}\right)}^{1/2},$$

where n=10 is the number of standards irradiated and *Q* is the reading of the individual TLD. The value of $\sigma_{D}$ is very uniform among the TLDs at approximately 6.5 cGy. Thus differences between TLD measurements and predicted values of less than $2\times \sigma_{D}=13$ cGy are not significant.

The Corvus and msnp predicted dose for each TLD capsule is based on the dose calculated by the treatment planning system at the center of the TLD capsule. A coordinate system transformation relating the coordinates used by the msnp code and that used by Corvus was established based on the position of three fiducial markers. This transformation was used to ensure that the dose was evaluated at the same location for msnp and Corvus.

**Figure 8:**
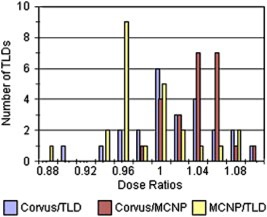
(Color) A histogram of the ratios of Corvus predicted dose values to TLD measured doses and to msnp predicted doses. The ratio of msnp predicted doses to TLD measured values is also shown. Given the spread in these distributions (3%−5%) it is not possible to distinguish them.

The average measured TLD dose is 191.7 cGy, the average dose predicted by Corvus is 192.0 cGy, and the average predicted dose by msnp is 186.9 cGy. The ratio of the Corvus average to the measured average is therefore approximately 1.002. A histogram of the dose ratios is shown in Fig. 8. The average value of the ratio of the Corvus calculated dose to the msnp calculated dose over the 24 locations of TLD placement is 1.028 with a standard deviation of 0.029. The average ratio of the Corvus predicted dose to the dose measured by the TLDs is 1.004 with a standard deviation of 0.045. The average ratio for the msnp predicted dose to the dose measured by the TLDs is 0.977 with a standard deviation of 0.045. It does not seem possible to distinguish the TLD measured dose from either the Corvus or msnp predictions within the accuracy of the TLD measurements (3%−5%).

## VII. DISCUSSION

Wang, Yorke, and Chui have compared the mskcc pencil beam algorithm against an adaptation of the EGS4 Monte Carlo code for 6 MV IMRT plans for lung and head and neck treatments.^10^ They have used patient CT data and have examined lung plans for five different patients. The pencil beam algorithm uses the equivalent path length method for inhomogeneity corrections. The location of our tumor volume is similar to that reported by Wang *et al*. in their study. All of their tumor volumes were in the right upper to middle medial lung and partially involved the mediastinum. These authors do not quote volumes for the CTV. Our CTV numerical volume is between their quoted GTV and PTV volumes. The lung plans employed 4–6 beams, but we are not told the beam directions. The results of their comparison are very similar to our results. These authors find significant discrepancies in the medial and posterior portions of the right lung. The discrepancies are predominantly in the high dose, low electron density regions. The average ratio of the Monte Carlo mean dose (PTV) to the pencil beam mean dose for the five patients is 0.965.

Laub, Bakai, and Nüsslin have carried out a study in which they have compared the IMRT dose distribution in an Alderson anthropomorphic phantom thorax computed by a pencil beam algorithm with Monte Carlo calculated doses and measured doses (TLD and film).^11^ The IMRT planning system is the KonRad IMRT system. Intensity modulated beams are delivered with the use of compensators which does present some complications. They have used five non‐coplanar 6 MV beams. The Monte Carlo code is an adaptation of EGS4. They found good agreement between Monte Carlo, pencil beam and measured doses. These authors do not find that the pencil beam algorithm overestimates the dose in the target volume. They offer three possible explanations for this: (i) a phantom was used instead of a patient data set, (ii) non‐coplanar beams suppress the differences, and (iii) intensity modulation. Reasons (i) and (iii) cannot be correct, as a phantom and intensity modulation have been used here. We cannot address reason (ii), as we have used coplanar beams.

Pawlicki and Ma describe a comparison between EGS4 based Monte Carlo calculations and Corvus for an upper thoracic target. Eight coplanar 4 MV beams are used for plan calculation^12^ The Monte Carlo calculations predict a 9% lower mean dose to the target than Corvus. Once again the pencil beam calculated doses are higher in the target than the EGS4 calculated doses because of electron transport out of the target into low density surrounding lung tissue. The Corvus pencil beam kernel is not laterally scaled to account for changes in lateral electron transport due to inhomogeneities.^13^

In Sec. II of this paper a caveat is mentioned regarding the true electron density of tumor tissue. In our study, the electron density of the PTV is based on the CT numbers inside the physician drawn (phony) tumor volume. The electron densities inside the lung portion of the tumor volume are therefore characteristic of normal lung tissue. Real lung tumors may have electron densities which are higher than surrounding normal lung tissue. As the Wang *et al*. study is based on patient CT data, it can be presumed that their PTV electron densities accurately characterize lung tumor tissue. The fact that the results of Wang *et al*. are similar to ours suggests that either the electron densities that we have used are similar to patient tumor densities or that the results are insensitive to this.

## VIII. CONCLUSION

We have made comparisons between calculated dose distributions and the measured dose distribution in the thorax of an anthropomorphic phantom for a lung treatment plan. The calculated dose distributions have been computed using the Corvus treatment planning software and by the msnp Monte Carlo code. The msnp computed and the measured dose values are in good agreement with Corvus values except in regions where the electron density is low and the dose is high. Corvus computes dose values which are up to 10% higher than msnp in these regions. The average Corvus calculated dose for the CTV is 5% higher than for the msnp computation. The clinical significance of these results is not clear to us.

These results are in good agreement with those of other workers who have compared a variety of pencil beam algorithms to EGS4 Monte Carlo calculations and measurement. The explanation suggested for this phenomenon is that pencil beam algorithms underestimate the degree of lateral electron transport out of low density regions and therefore overestimate the dose.^10^

## ACKNOWLEDGMENTS

We thank Dr. Jim Fontanesi for drawing the CTV. We also thank Dr. Jay Burmeister for advice.
